# Supplementary material for: Pyruvate Dehydrogenase Kinase 1 inhibition mediated oxidative phosphorylation enhancement in cartilage promotes osteoarthritis progression
Source: BMC Musculoskelet Disord. 2023 Jul 20;24:597. doi: 10.1186/s12891-023-06585-6 (PMC10357736; doi:10.1186/s12891-023-06585-6)
Supplement: Supplementary file 1 — Additional file 1. [file 12891_2023_6585_MOESM1_ESM.zip › MF.Gsea.1653627316242/GOMF_ATP_DEPENDENT_ACTIVITY_ACTING_ON_RNA.html]

Details for gene set GOMF\_ATP\_DEPENDENT\_ACTIVITY\_ACTING\_ON\_RNA[GSEA]

|  || Dataset | 55457\_collapsed\_to\_symbols.cls.cls #OA\_versus\_normal.cls.cls #OA\_versus\_normal\_repos |
| Phenotype | cls.cls#OA\_versus\_normal\_repos |
| Upregulated in class | normal |
| GeneSet | GOMF\_ATP\_DEPENDENT\_ACTIVITY\_ACTING\_ON\_RNA |
| Enrichment Score (ES) | -0.50520015 |
| Normalized Enrichment Score (NES) | -2.0407846 |
| Nominal p-value | 0.0 |
| FDR q-value | 0.011963081 |
| FWER p-Value | 0.034 |
Table: GSEA Results Summary

  

Fig 1: Enrichment plot: GOMF\_ATP\_DEPENDENT\_ACTIVITY\_ACTING\_ON\_RNA      
 Profile of the Running ES Score & Positions of GeneSet Members on the Rank Ordered List

  

| SYMBOL | TITLE | RANK IN GENE LIST | RANK METRIC SCORE | RUNNING ES | CORE ENRICHMENT || 1 | DDX23 | DEAD-box helicase 23 [Source:HGNC Symbol;Acc:HGNC:17347] | 913 | 0.538 | -0.0485 | No |
| 2 | DHX29 | DExH-box helicase 29 [Source:HGNC Symbol;Acc:HGNC:15815] | 1012 | 0.515 | -0.0345 | No |
| 3 | DDX60 | DExD/H-box helicase 60 [Source:HGNC Symbol;Acc:HGNC:25942] | 1385 | 0.445 | -0.0448 | No |
| 4 | DDX6 | DEAD-box helicase 6 [Source:HGNC Symbol;Acc:HGNC:2747] | 1687 | 0.401 | -0.0514 | No |
| 5 | MTREX | Mtr4 exosome RNA helicase [Source:HGNC Symbol;Acc:HGNC:18734] | 2138 | 0.346 | -0.0720 | No |
| 6 | IFIH1 | interferon induced with helicase C domain 1 [Source:HGNC Symbol;Acc:HGNC:18873] | 2204 | 0.338 | -0.0628 | No |
| 7 | DHX57 | DExH-box helicase 57 [Source:HGNC Symbol;Acc:HGNC:20086] | 2262 | 0.331 | -0.0534 | No |
| 8 | DDX4 | DEAD-box helicase 4 [Source:HGNC Symbol;Acc:HGNC:18700] | 2788 | 0.271 | -0.0829 | No |
| 9 | DDX50 | DExD-box helicase 50 [Source:HGNC Symbol;Acc:HGNC:17906] | 3167 | 0.234 | -0.1025 | No |
| 10 | DDX1 | DEAD-box helicase 1 [Source:HGNC Symbol;Acc:HGNC:2734] | 3305 | 0.221 | -0.1039 | No |
| 11 | DHX58 | DExH-box helicase 58 [Source:HGNC Symbol;Acc:HGNC:29517] | 4130 | 0.143 | -0.1621 | No |
| 12 | DHX40 | DEAH-box helicase 40 [Source:HGNC Symbol;Acc:HGNC:18018] | 4358 | 0.124 | -0.1746 | No |
| 13 | UPF1 | UPF1 RNA helicase and ATPase [Source:HGNC Symbol;Acc:HGNC:9962] | 4434 | 0.118 | -0.1754 | No |
| 14 | DDX19B | DEAD-box helicase 19B [Source:HGNC Symbol;Acc:HGNC:2742] | 4500 | 0.113 | -0.1758 | No |
| 15 | DDX28 | DEAD-box helicase 28 [Source:HGNC Symbol;Acc:HGNC:17330] | 4602 | 0.105 | -0.1792 | No |
| 16 | DDX47 | DEAD-box helicase 47 [Source:HGNC Symbol;Acc:HGNC:18682] | 4789 | 0.088 | -0.1900 | No |
| 17 | DDX41 | DEAD-box helicase 41 [Source:HGNC Symbol;Acc:HGNC:18674] | 5004 | 0.073 | -0.2037 | No |
| 18 | DHX8 | DEAH-box helicase 8 [Source:HGNC Symbol;Acc:HGNC:2749] | 5531 | 0.030 | -0.2434 | No |
| 19 | DDX46 | DEAD-box helicase 46 [Source:HGNC Symbol;Acc:HGNC:18681] | 5744 | 0.012 | -0.2594 | No |
| 20 | RAD54B | RAD54 homolog B [Source:HGNC Symbol;Acc:HGNC:17228] | 5753 | 0.012 | -0.2595 | No |
| 21 | BRIP1 | BRCA1 interacting helicase 1 [Source:HGNC Symbol;Acc:HGNC:20473] | 5885 | 0.003 | -0.2696 | No |
| 22 | DDX58 | DExD/H-box helicase 58 [Source:HGNC Symbol;Acc:HGNC:19102] | 6480 | -0.041 | -0.3141 | No |
| 23 | SNRNP200 | small nuclear ribonucleoprotein U5 subunit 200 [Source:HGNC Symbol;Acc:HGNC:30859] | 7249 | -0.099 | -0.3698 | No |
| 24 | DDX42 | DEAD-box helicase 42 [Source:HGNC Symbol;Acc:HGNC:18676] | 7447 | -0.114 | -0.3803 | No |
| 25 | EIF4A3 | eukaryotic translation initiation factor 4A3 [Source:HGNC Symbol;Acc:HGNC:18683] | 7646 | -0.130 | -0.3903 | No |
| 26 | AQR | aquarius intron-binding spliceosomal factor [Source:HGNC Symbol;Acc:HGNC:29513] | 7654 | -0.131 | -0.3853 | No |
| 27 | DDX49 | DEAD-box helicase 49 [Source:HGNC Symbol;Acc:HGNC:18684] | 7945 | -0.151 | -0.4016 | No |
| 28 | DDX18 | DEAD-box helicase 18 [Source:HGNC Symbol;Acc:HGNC:2741] | 7996 | -0.154 | -0.3990 | No |
| 29 | DHX30 | DExH-box helicase 30 [Source:HGNC Symbol;Acc:HGNC:16716] | 8111 | -0.164 | -0.4010 | No |
| 30 | SKIV2L | Ski2 like RNA helicase [Source:HGNC Symbol;Acc:HGNC:10898] | 8395 | -0.185 | -0.4153 | No |
| 31 | DDX25 | DEAD-box helicase 25 [Source:HGNC Symbol;Acc:HGNC:18698] | 8815 | -0.218 | -0.4387 | No |
| 32 | EIF4A2 | eukaryotic translation initiation factor 4A2 [Source:HGNC Symbol;Acc:HGNC:3284] | 9251 | -0.252 | -0.4620 | No |
| 33 | DDX24 | DEAD-box helicase 24 [Source:HGNC Symbol;Acc:HGNC:13266] | 9588 | -0.282 | -0.4764 | No |
| 34 | YTHDC2 | YTH domain containing 2 [Source:HGNC Symbol;Acc:HGNC:24721] | 9872 | -0.309 | -0.4854 | No |
| 35 | DHX32 | DEAH-box helicase 32 (putative) [Source:HGNC Symbol;Acc:HGNC:16717] | 10127 | -0.334 | -0.4912 | Yes |
| 36 | DDX51 | DEAD-box helicase 51 [Source:HGNC Symbol;Acc:HGNC:20082] | 10133 | -0.334 | -0.4775 | Yes |
| 37 | DHX35 | DEAH-box helicase 35 [Source:HGNC Symbol;Acc:HGNC:15861] | 10215 | -0.342 | -0.4695 | Yes |
| 38 | IGHMBP2 | immunoglobulin mu DNA binding protein 2 [Source:HGNC Symbol;Acc:HGNC:5542] | 10248 | -0.346 | -0.4574 | Yes |
| 39 | DHX15 | DEAH-box helicase 15 [Source:HGNC Symbol;Acc:HGNC:2738] | 10498 | -0.372 | -0.4612 | Yes |
| 40 | DHX9 | DExH-box helicase 9 [Source:HGNC Symbol;Acc:HGNC:2750] | 10627 | -0.386 | -0.4550 | Yes |
| 41 | DDX10 | DEAD-box helicase 10 [Source:HGNC Symbol;Acc:HGNC:2735] | 11029 | -0.434 | -0.4680 | Yes |
| 42 | G3BP1 | G3BP stress granule assembly factor 1 [Source:HGNC Symbol;Acc:HGNC:30292] | 11117 | -0.445 | -0.4561 | Yes |
| 43 | DDX21 | DExD-box helicase 21 [Source:HGNC Symbol;Acc:HGNC:2744] | 11120 | -0.446 | -0.4375 | Yes |
| 44 | TDRD12 | tudor domain containing 12 [Source:HGNC Symbol;Acc:HGNC:25044] | 11283 | -0.469 | -0.4304 | Yes |
| 45 | DDX39A | DExD-box helicase 39A [Source:HGNC Symbol;Acc:HGNC:17821] | 11359 | -0.480 | -0.4161 | Yes |
| 46 | DDX56 | DEAD-box helicase 56 [Source:HGNC Symbol;Acc:HGNC:18193] | 11386 | -0.484 | -0.3978 | Yes |
| 47 | DDX52 | DExD-box helicase 52 [Source:HGNC Symbol;Acc:HGNC:20038] | 11484 | -0.499 | -0.3844 | Yes |
| 48 | DDX17 | DEAD-box helicase 17 [Source:HGNC Symbol;Acc:HGNC:2740] | 11716 | -0.535 | -0.3799 | Yes |
| 49 | DDX19A | DEAD-box helicase 19A [Source:HGNC Symbol;Acc:HGNC:25628] | 11821 | -0.553 | -0.3647 | Yes |
| 50 | DHX38 | DEAH-box helicase 38 [Source:HGNC Symbol;Acc:HGNC:17211] | 11860 | -0.562 | -0.3441 | Yes |
| 51 | DDX54 | DEAD-box helicase 54 [Source:HGNC Symbol;Acc:HGNC:20084] | 11969 | -0.587 | -0.3279 | Yes |
| 52 | DHX34 | DExH-box helicase 34 [Source:HGNC Symbol;Acc:HGNC:16719] | 12040 | -0.603 | -0.3080 | Yes |
| 53 | SUPV3L1 | Suv3 like RNA helicase [Source:HGNC Symbol;Acc:HGNC:11471] | 12087 | -0.616 | -0.2857 | Yes |
| 54 | EIF4A1 | eukaryotic translation initiation factor 4A1 [Source:HGNC Symbol;Acc:HGNC:3282] | 12111 | -0.623 | -0.2613 | Yes |
| 55 | DDX31 | DEAD-box helicase 31 [Source:HGNC Symbol;Acc:HGNC:16715] | 12288 | -0.672 | -0.2468 | Yes |
| 56 | DDX27 | DEAD-box helicase 27 [Source:HGNC Symbol;Acc:HGNC:15837] | 12359 | -0.695 | -0.2231 | Yes |
| 57 | DHX16 | DEAH-box helicase 16 [Source:HGNC Symbol;Acc:HGNC:2739] | 12414 | -0.712 | -0.1974 | Yes |
| 58 | DDX11 | DEAD/H-box helicase 11 [Source:HGNC Symbol;Acc:HGNC:2736] | 12607 | -0.803 | -0.1786 | Yes |
| 59 | DDX3Y | DEAD-box helicase 3 Y-linked [Source:HGNC Symbol;Acc:HGNC:2699] | 12728 | -0.890 | -0.1506 | Yes |
| 60 | DDX39B | DExD-box helicase 39B [Source:HGNC Symbol;Acc:HGNC:13917] | 12783 | -0.941 | -0.1153 | Yes |
| 61 | DDX3X | DEAD-box helicase 3 X-linked [Source:HGNC Symbol;Acc:HGNC:2745] | 12788 | -0.949 | -0.0757 | Yes |
| 62 | DDX43 | DEAD-box helicase 43 [Source:HGNC Symbol;Acc:HGNC:18677] | 12800 | -0.969 | -0.0359 | Yes |
| 63 | DDX5 | DEAD-box helicase 5 [Source:HGNC Symbol;Acc:HGNC:2746] | 12834 | -1.038 | 0.0051 | Yes |
Table: GSEA details [plain text format]

  

Fig 2: GOMF\_ATP\_DEPENDENT\_ACTIVITY\_ACTING\_ON\_RNA      
 Blue-Pink O' Gram in the Space of the Analyzed GeneSet

  

Fig 3: GOMF\_ATP\_DEPENDENT\_ACTIVITY\_ACTING\_ON\_RNA: Random ES distribution      
 Gene set null distribution of ES for **GOMF\_ATP\_DEPENDENT\_ACTIVITY\_ACTING\_ON\_RNA**
